# Body mass index impacts ectopic pregnancy during in vitro fertilization: an analysis of 42,362 clinical pregnancy cycles

**DOI:** 10.1186/s12958-023-01146-x

**Published:** 2023-10-31

**Authors:** Xiaofei Ge, Keyan Wang, Yingpu Sun, Zhiqin Bu

**Affiliations:** 1 Reproductive Medical Center, the First Affiliated Hospital of Zhengzhou University, 1# Jianshe East Road, Zhengzhou, China; 2 Henan Institute of Medical and Pharmaceutical Sciences, Zhengzhou University, Zhengzhou, 450052 China

**Keywords:** Female BMI, Ectopic pregnancy, IVF/ICSI, Embryo transfer

## Abstract

**Purpose:**

This large, single-center, retrospective cohort study was aimed to explore the effect of female body mass index (BMI) on ectopic pregnancy (EP) following fresh and frozen-thawed embryo transfers (ET).

**Methods:**

A total of 27,600 pregnancies after fresh ET and 14,762 pregnancies after frozen-thawed ET were included between January 2010 to June 2022. Women were divided into three groups based on BMI according to the Working Group on Obesity in China (WGOC), International Life Sciences Institute (ILSI): underweight (BMI < 18.5 kg/m^2^), normal weight (BMI, 18.5–23.9 kg/m^2^), and overweight or obesity (≥ 24 kg/m^2^). Compare EP rates among BMI categories in fresh and frozen-thawed ET cycles respectively. Multivariate logistic regression analyses were used to investigate the association between female BMI and EP.

**Results:**

The overall EP rates in fresh, and frozen thawed transfer cycles were 2.43% (672/27,600) and 2.82% (417/14,762), respectively. In fresh ET cycles, underweight women yielded a significantly higher EP rate than those with normal and excess weight (3.29% vs. 2.29% vs. 2.54%, *P* = 0.029). But EP rates did not differ among the three BMI groups (2.72% vs. 2.76% vs. 2.96%, *P* = 0.782) in frozen-thawed ET cycles. In fresh ET cycles, after adjusting for potential confounding factors, no significant association was found between female BMI and EP occurrence (adjusted OR: 0.98, 95% CI 0.70–1.37, *P* = 0.894, for BMI 18.5–23.9 kg/m^2^; adjusted OR: 0.89, 95% CI 0.75–1.06, *P* = 0.205, for BMI ≥ 24 kg/m^2^. Reference = BMI < 18.5 kg/m^2^).

**Conclusion(s):**

Female BMI did not affect the occurrence of ectopic pregnancy in either fresh or frozen-thawed embryo transfer cycles.

## Introduction

Ectopic pregnancy (EP), which mainly includes fallopian tube pregnancy, cornual pregnancy, cervical pregnancy, and ovarian pregnancy, is one of the potentially life-threatening conditions [1, 2]. In pregnancies after natural conception, the incidence of this early pregnancy complication varies from 1 to 2% [3]. However, the rate of EP in assisted reproduction techniques (ART), especially in in vitro fertilization (IVF) and embryo transfer (ET) treatment cycles, reaches 1.4-8.6% [4]. During spontaneous pregnancy, it is known that EP is highly associated with tubal surgery history, pelvic inflammatory disease. However, tubal factor infertility is one of the most common reasons for women undergoing IVF-ET treatment. Therefore, it is reasonable that the incidence of EP in IVF-ET cycles is much higher than that in natural conception. Currently, several large cohort studies consistently indicated that other factors, including stage of embryos transferred (cleavage stage or blastocyst stage), high estrogen levels during ovarian stimulation, and endometrial thickness, had an impact on EP rate in IVF-ET cycles [5–7]. In addition, many other studies also demonstrated that other potential factors including ovarian stimulation protocols, number of embryos transferred, and type of embryos (fresh or frozen-thawed) were associated with EP rate [8–10].

Body mass index (BMI) is believed to be associated with both pregnancy outcomes and obstetrical compilations during IVF treatment. Having a high BMI before IVF treatment increased the risk of embryo implantation failure and spontaneous miscarriage in infertile patients [11, 12]. Moreover, overweight and obesity were also risk factors for gestational diabetes, preterm birth, low birth weight, postpartum infection, and other obstetrical and prenatal complications [13, 14].

Recently, a few studies have examined the association of maternal BMI with EP during IVF. In 2016, a nationwide database study showed that, in both luteal Gonadotropin-releasing hormone (GnRH) agonist and GnRH antagonist protocols, there was an significant increase of EP in patients with BMI ≥ 30 kg/m^2^ in comparison with non-obese patients (BMI < 30 kg/m^2^) [8].However, another large sample study reported that the rate of EP was significantly increased in the low BMI group (< 18.5 kg/m^2^), but not in the high BMI group (≥ 25 kg/m^2^), indicating that low BMI was associated with an increased risk of EP [15].

BMI is a critical parameter for IVF treatment. The starting dosage of gonadotrophins for ovarian stimulation is usually based on female BMI before IVF. More importantly, BMI also influences our decision in embryo transfer strategy to reduce the risk of ovarian hyperstimulation syndrome (OHSS) in fresh cycles, as lean women are companied with higher estrogen level and will benefit from selective single blastocyst transfer. As mentioned above, estrogen level and blastocyst transfer are both critical factors affecting EP. Thus, it is necessary to adjust for confounding factors when exploring the impact of BMI on EP.

Understanding the impact of BMI on EP during IVF treatment may be useful for predicting results and lead to the development of giving proper suggestions to patients. To answer the question of whether underweight and overweight/obesity predict EP, we performed this large cohort study using data from women undergoing fresh or frozen- thawed embryo transfers in our center from 2010 to 2022.

## Materials and methods

### Study design and population

This retrospective study included all IVF/intracytoplasmic sperm injection (ICSI)-ET cycles with outcomes reported as clinical pregnancy (including clinical intrauterine, ectopic, or heterotopic pregnancy) from January 2010 to June 2022 at the Reproductive Medicine Center of the First Affiliated Hospital of Zhengzhou University. Data collection was from the Clinical Reproductive Medicine Management System/Electronic Medical Record Cohort Database (CCRM/EMRCD) of our center. This study was approved by the Ethics Review Committee of the hospital and written informed consent was waived due to the retrospective nature of the study.

**Fig. 1:**
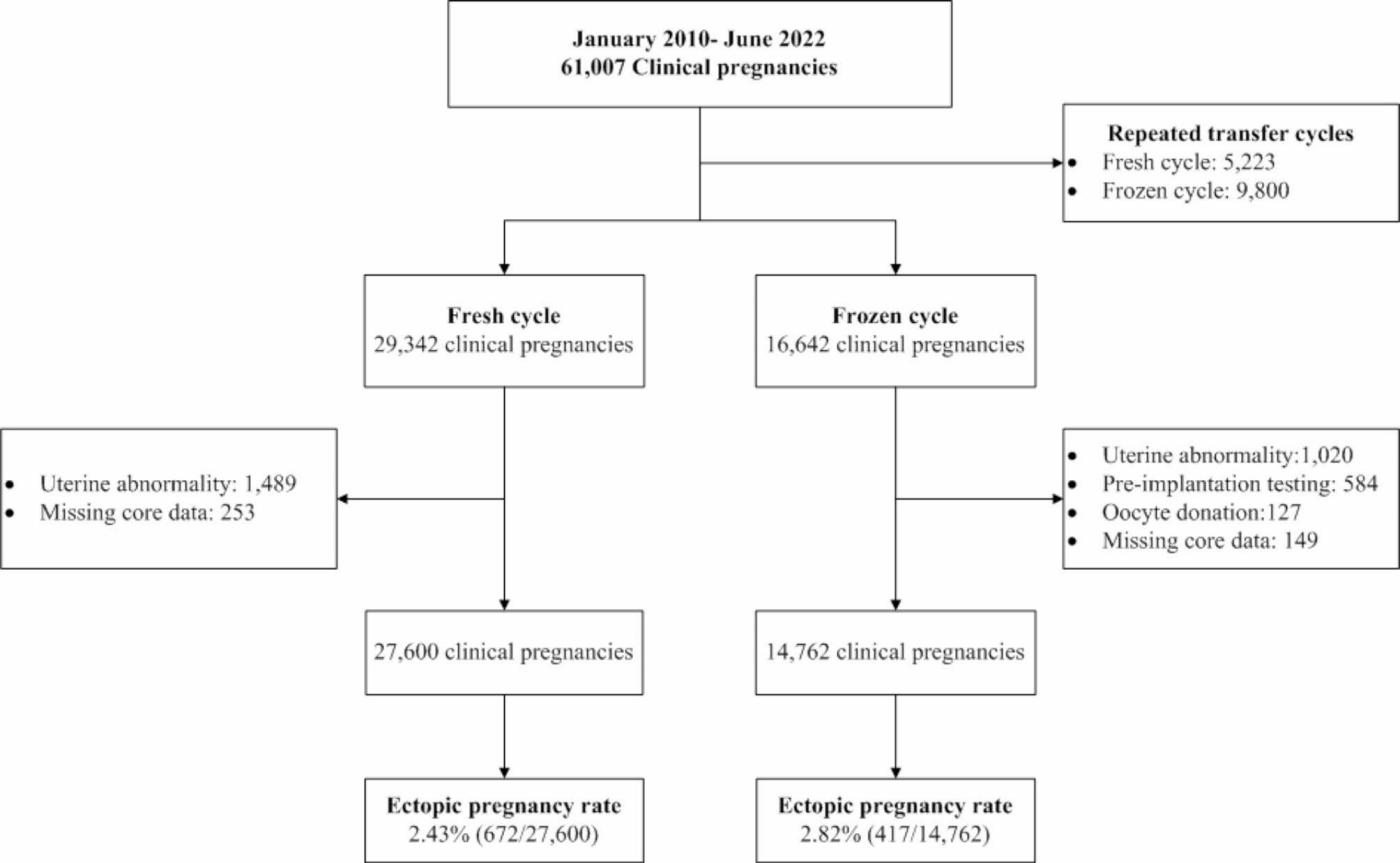
Flowchart of the retrospective cohort study

All patients underwent routine uterine ultrasound and hysteroscopy before ovarian stimulation. To avoid the interaction caused by repeated cycles, only patients between the ages of 20 and 45 years who had their first autologous fresh/frozen-thawed embryo transfer (FET) cycles were included. The exclusion criteria were as follows: preimplantation genetic diagnosis/screening cycles; patients with untreated hydrosalpinx; patients with uterine abnormalities (uterine malformation; uterine fibroids ≥ 3 cm in diameter or compressing the endometrium; endometrial polyps; intrauterine adhesion); cycles with incomplete core data. The definition of intrauterine pregnancy (IUP) and EP was elaborated in our previous study [7]. Heterotopic pregnancy was also classified as the EP. A detailed flow chart of sample selection is shown in Fig. 1.

Maternal BMI was derived from measured height and weight recorded at the beginning of ovarian stimulation or endometrial preparation. BMI was categorized into three groups: underweight (< 18.5 kg/m^2^), normal weight (18.5–23.9 kg/m^2^), overweight and obesity (≥ 24 kg/m^2^). These criteria were from Working Group on Obesity in China (WGOC), International Life Sciences Institute (ILSI) [16]. The diagnosis of tubal infertility has also been described previously [7]. Cleavage stage embryo refers to embryo at day 3. Blastocyst stage includes embryos at day 5 or day 6. Endometrial thickness was measured on trigger day in fresh ET cycles, or on day of ovulation/progesterone administration in frozen thawed ET (FET) cycles. Patients were divided into thin (< 7 mm), medium (8–13 mm), and thick (≥ 14 mm) groups according to endometrial thickness. Peak estrogen level was measured on trigger day in fresh embryo transfer cycles.

### ART protocols

In fresh cycles, ovarian stimulation protocols were carried out depending on female age and ovarian reserve. During the cycle, follicle growth was regularly monitored by transvaginal ultrasound and serum sex hormone levels, and the gonadotropin (Gn) dose was adjusted accordingly. When at least two follicles reached a diameter of 16 mm or wider, oocyte maturation was triggered by human chorionic gonadotropin (hCG). Transvaginal ultrasound-guided oocyte retrieval, fertilization, and embryo culture were performed as described elsewhere [17]. Fresh cleavage embryos or blastocysts were selected for transfer according to the patient’s condition and embryo quality. Luteal support was started on the day of oocyte retrieval by using oral progesterone and daily transvaginal progesterone.

In FET cycles, the protocols for endometrial preparation included natural cycles and artificial cycles, which were mainly selected based on patients’ menstruation and their doctor’s experience. After vitrified embryos being warmed, ET was conducted under ultrasound guidance. The details of protocols were published previously [18] .

All patients were followed up and luteal support was continued if the serum hCG concentration was > 50 IU/L 14 days after ET. Transvaginal ultrasound was performed 5 weeks after ET and the definition of IUP and EP was described in detail previously [7].

### Statistical analysis

Data were examined for normal distribution, and appropriate tests were applied. The data were presented as mean ± standard deviation (SD) for normal distribution, and analyzed by Student’s *t* test or one-way analysis of variance. For non-normal distribution data, they were shown as medium (25th, 75th ), and nonparametric test (Kruskal-Wallis test) was performed for comparation. Chi-square test was used to detect difference between categorical variables.

Firstly, possible factors affecting EP were screened in both fresh and frozen-thawed ET cycles. Then, multivariate logistic regression analysis was conducted to investigate the association between female BMI and EP after adjusting potential confounding risk factors. In fresh ET cycles, adjusted factors were BMI, infertility type, tubal factor, peak estrogen level, endometrial thickness, type of embryos, and number of embryos transferred. In FET cycles, adjusted factors were BMI, tubal factor, endometrial thickness, type of embryos, and number of embryos transferred. Statistical analysis was performed with SPSS (Statistical Package for Social Science, SPSS Inc, Chicago, IL, USA) version 21.0. A *P* < 0.05 was considered statistically significant.

## Results

### Characteristics of the study cohort

From January 2010 to June 2022, a total of 61,007 cycles were reported as clinical pregnancy after fresh, or frozen-thawed ET. After excluding repeat cycles and patients with exclusion criteria, 27,600 pregnant cycles following fresh ET and 14,762 pregnant cycles after FET were included in the final analysis. In total, there were 672 and 471 ectopic pregnancies in fresh, and frozen-thawed ET cycles, respectively. The overall EP rate was 2.57% (1,089/42,362). EP rate was 2.43% (672/27,600) and 2.82% (417/14,762) in fresh, and frozen-thawed ET cycles, respectively (Fig. 1).

### Comparison of differences among three BMI groups

Table 1 showed the EP rates in different groups. In fresh ET cycles, EP rates were comparable among patients with different age (< 35 years old, or ≥ 35 years old), and different ovarian stimulation protocols (agonist, antagonist, or others). However, underweight patients yielded a significantly higher EP rate compared with normal and overweight/obesity women (3.29% vs. 2.29% vs. 2.54%; *P* = 0.029). Moreover, the EP rate was also higher in patients with secondary infertility (compared with primary infertility), with tubal infertility (compared with non-tubal infertility), with cleavage stage embryo transfer (compared with blastocyst transfer).

**Table 1:** Ectopic pregnancy rate in fresh embryo transfer cycles and in frozen-thawed embryo transfer cycles

	Fresh embryo transfer					Frozen-thawed embryo transfer			
	CP	EP	EP rate	*P* value		CP	EP	EP rate	*P* value
Total No.	27,600	672	2.43%			14,762	417	2.82%	
Age (year)									
< 35	22,914	557	2.43%	0.925		12,064	342	2.83%	0.876
≥ 35	4686	115	2.45%			2698	75	2.78%	
BMI (kg/m^2^)									
< 18.5	1703	56	3.29%			883	24	2.72%	
18.5–23.9	16,840	386	2.29%	0.029		8840	244	2.76%	0.782
≥ 24	9057	230	2.54%			5039	149	2.96%	
Infertility type									
Primary	15,265	343	2.25%	0.024		6779	173	2.56%	0.065
Secondary	12,335	329	2.67%			7983	244	3.06%	
Tubal factor existed									
Yes	11,075	402	3.63%	< 0.001		5902	207	3.51%	< 0.001
No	16,525	270	1.63%			8860	210	2.37%	
Ovarian stimulation protocol									
Agonist	27,045	658	2.43%			/			
Antagonist	250	5	2.00%	0.763		/			
Others^*^	305	9	2.95%			/			
Peak Estrogen level (pg/ml)									
< 2500	9806	216	2.20%			/			
[2500–5000)	12,092	286	2.37%	0.011		/			
[5000–7500)	4104	116	2.83%			/			
≥ 7500	1598	54	3.38%			/			
Endometrial preparation Protocol									
Natural cycle	/					5204	133	2.56%	0.145
Artificial cycle	/					9558	284	2.97%	
Endometrial thickness (mm)^+^									
Thin (< 7)	385	21	5.45%			357	23	6.44%	
Medium (8–13)	17,901	480	2.68%	< 0.001		7624	238	3.12%	< 0.001
Thick (≥ 14)	8420	147	1.75%			607	9	1.48%	
Type of embryos									
Cleavage	22,222	582	2.62%	< 0.001		6503	241	3.71%	< 0.001
Blastocyst	5378	90	1.67%			8259	176	2.13%	
No. of embryos transferred									
1	6793	125	1.84%			6343	139	2.19%	
2	20,328	537	2.64%	0.001		7448	229	3.07%	< 0.001
3	479	10	2.09%			971	49	5.05%	

*Note*: * other protocols include mild stimulation, natural cycle, and short protocol; BMI: body mass index; CP: clinical pregnancy; EP: ectopic pregnancy;

^+^ missing data existed; endometrial thickness was measured on trigger day in fresh cycle, or on day of ovulation/progesterone administration in frozen thawed cycle

In addition, the EP rates increased with number of embryos transferred, while decreased with endometrial thickness. Interestingly, there seemed to be a positive correlation between EP rates and peak estrogen levels. It was < 2.5% in patients with estrogen < 5000 pg/ml, and 3.38% in patients with high estrogen level (≥ 7500 pg/ml). In frozen-thawed embryo transfer cycles, the situation was similar with that in fresh embryo transfer cycles. However, the EP rates did not differ among different BMI categories (2.72% vs. 2.76% vs. 2.96%; *P* = 0.782).

Basic parameters and EP rates in fresh ET cycles with different BMI were compared in Table 2. Besides EP rate, patients’ basic demographic characteristics were different among underweight, normal weight, and overweight/obesity groups. Importantly, peak estrogen level was significantly higher in underweight patients when compared with that in the other two groups (3787 pg/ml vs. 3324 pg/ml vs. 2676 pg/ml; *P* < 0.001).

**Table 2:** Basic parameters and ectopic pregnancy rates in fresh embryo transfer cycles with different body mass index (kg/m^2^)

	< 18.5	18.5–23.9	≥ 24	*P* value
No.	1703	16,840	9057	
Age (year)	28.93 ± 3.80	30.26 ± 4.30	30.74 ± 4.55	< 0.001
BMI (kg/m^2^)	17.72 ± 0.66	21.35 ± 1.47	26.56 ± 2.12	< 0.001
Infertility type				< 0.001
Primary	1097 (64.42%)	9437 (56.04%)	4731 (52.24%)	
Secondary	606 (35.58%)	7403 (43.96%)	4326 (47.76%)	
Tubal factor existed				< 0.001
Yes	772 (45.33%)	6740 (40.02%)	3463 (38.24%)	
No	931 (54.67%)	10,100 (59.98%)	5594 (61.76%)	
Peak Estrogen (pg/ml), Mean (25th ,75th )	3787 (2513, 5356)	3324 (2191, 4891)	2676 (1752, 4034)	< 0.001
Endometrial thickness (mm)	12.31 ± 2.45	12.42 ± 2.59	12.40 ± 2.61	< 0.001
Type of embryos				< 0.001
Cleavage	1424 (83.62%)	13,807 (81.99%)	6991 (77.19%)	
Blastocyst	279 (16.38%)	3033 (18.01%)	2066 (22.81%)	
No. of embryos transferred	1.79 ± 0.43	1.79 ± 0.45	1.73 ± 0.48	
1	367 (21.55%)	3818 (22.67%)	2608 (28.80%)	
2	1322 (77.63%)	12,694 (75.38%)	6312 (69.69%)	< 0.001
3	14 (0.82%)	328 (1.95%)	137 (1.51%)	
Ectopic pregnancy, n (%)	56 (3.29%)	386 (2.29% )	230 (2.54%)	0.029

*Note* Data were shown as mean ± standard deviation unless otherwise indicated; BMI, body mass index

**Fig. 2:**
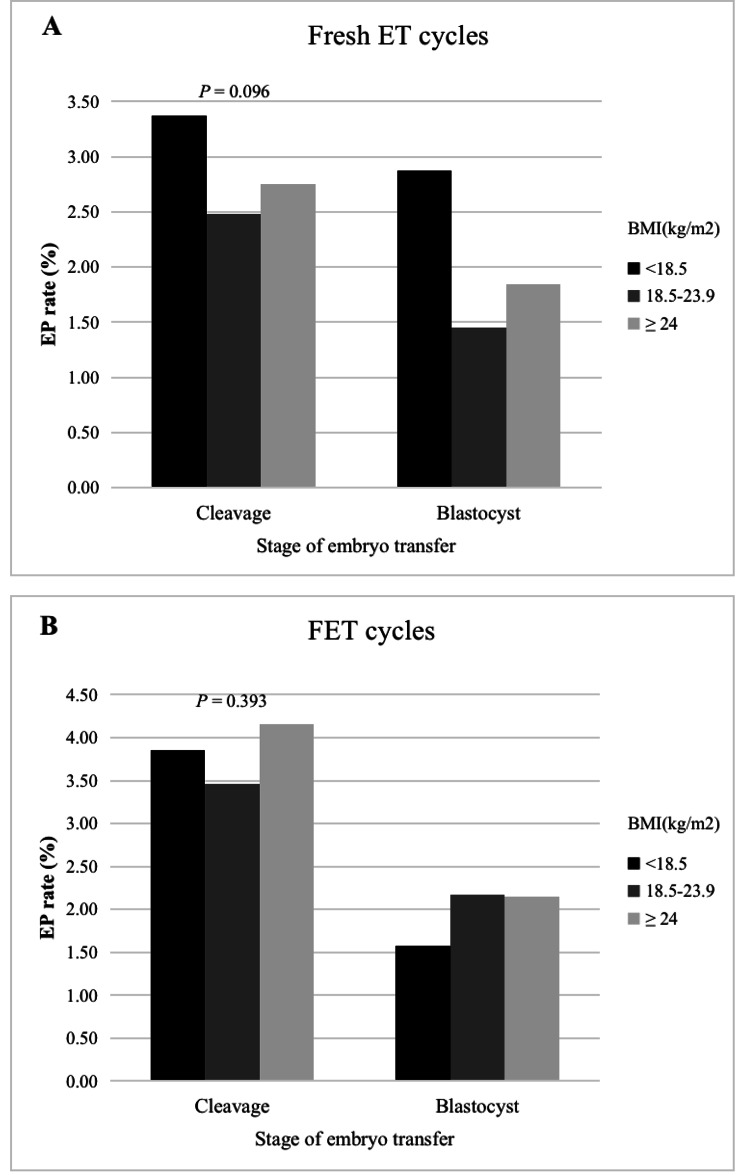
Ectopic pregnancy rates according to female body mass index classification and the stage of embryo transfer. (**A**) EP rates in different BMI groups in fresh ET cycles. (**B**) EP rates in different BMI groups in FET cycles. *Note*: BMI: body mass index; EP: ectopic pregnancy; FET: frozen-thawed embryo transfer; EP rate given as n = ectopic pregnancy/N = clinical pregnancy. Statistical differences analyzed by chi-squared test

In Table 3, it was shown that baseline was not comparable among these three BMI groups, either. However, the ectopic pregnancy rate was similar. The ectopic pregnancy rate was also further compared in cleavage embryo and blastocyst embryo transfer cycles. As shown in Fig. 2, in fresh embryo transfer cycles, the ectopic pregnancy rate was comparable among three BMI groups in both cleavage and blastocyst transfers (Cleavage stage: 3.37% vs. 2.48% vs. 2.75%, *P* = 0.096; Blastocyst stage: 2.87% vs. 1.45% vs. 1.84%; *P* = 0.159). In frozen thawed embryo transfer cycles, the ectopic pregnancy rate was also similar among three BMI groups irrespective of embryo stage (Cleavage stage: 3.85% vs. 3.46% vs. 4.16%, *P* = 0.393; Blastocyst stage: 1.58% vs. 2.17% vs. 2.15%; *P* = 0.714).

**Table 3:** Basic parameters and ectopic pregnancy rate in frozen-thawed embryo transfer cycles with different body mass index (kg/m^2^)

	< 18.5	18.5–23.9	≥ 24	*P* value
No.	883	8840	5039	
Age (year)	29.03 ± 4.11	30.50 ± 4.46	31.03 ± 4.84	< 0.001
BMI (kg/m^2^)	17.68 ± 0.67	21.40 ± 1.47	26.69 ± 2.22	< 0.001
Infertility type				
Primary	496 (56.17%)	4122 (46.63%)	2161 (42.89%)	< 0.001
Secondary	387 (43.83%)	4718 (53.37%)	2878 (57.11%)	
Tubal factor existed				
Yes	383 (43.37%)	3587 (40.58%)	1932 (38.34%)	< 0.001
No	500 (56.63%)	5253 (59.42%)	3107 (61.66%)	
Endometrial preparation Protocol				
Natural cycle	350 (39.64%)	3415 (38.63%)	1439 (28.56%)	< 0.001
Artificial cycle	533 (60.36%)	5425 (61.37%)	3600 (71.44%)	
Endometrial thickness (mm)	10.50 ± 1.95	10.39 ± 1.99	10.30 ± 2.03	0.034
Type of embryos				
Cleavage	441 (49.94%)	4043 (45.74%)	2019 (40.07%)	< 0.001
Blastocyst	442 (50.06%)	4797 (54.26%)	3020 (59.93%)	
No. of embryos transferred	1.72 ± 0.58	1.65 ± 0.60	1.59 ± 0.60	< 0.001
1	310 (35.11%)	3666 (41.47%)	2367 (46.97%)	
2	512 (57.98%)	4558 (51.56%)	2378 (47.19%)	< 0.001
3	61 (6.91%)	616 (6.97%)	294 (5.84%)	
Ectopic pregnancy, n (%)	24 (2.72%)	244 (2.76%)	149 (2.96%)	0.782

*Note* Data were shown as mean ± standard deviation unless otherwise indicated; BMI, body mass index

### Relationship between female BMI and EP

In Table 4, multivariate logistic regression analysis was performed to explore risk factors for ectopic pregnancy. In fresh embryo transfer cycles, secondary infertility, tubal infertility, elevated estrogen level, thin endometrial thickness, and cleavage stage embryo transfer were risk factors for ectopic pregnancy. However, female BMI was not associated with ectopic pregnancy (adjusted OR: 0.98, *P* = 0.894, for BMI 18.5–23.9 kg/m^2^; adjusted OR: 0.89, *P* = 0.205, for BMI ≥ 24 kg/m^2^. Reference = BMI < 18.5 kg/m^2^). In FET cycles, only thin endometrial thickness, and cleavage stage embryo transfer were risk factors for ectopic pregnancy. Female BMI was not predictable for ectopic pregnancy, either (adjusted OR: 1.15, *P* = 0.513, for BMI 18.5–23.9 kg/m^2^; adjusted OR: 1.24, *P* = 0.367, for BMI ≥ 24 kg/m^2^. Reference = BMI < 18.5 kg/m^2^).

**Table 4:** Multivariate logistic regression analysis of risk factors for ectopic pregnancy

	Fresh embryo transfer			Frozen-thawed embryo transfer	
	Adjusted OR (95% CI)	*P*		Adjusted OR (95% CI)	*P*
BMI (kg/m^2^)					
< 18.5	Reference			Reference	
18.5–23.9	0.98 (0.70–1.37)	0.894		1.15 (0.89–2.05)	0.513
≥ 24	0.89 (0.75–1.06)	0.205		1.24 (0.94–2.65)	0.367
Infertility type				/	
Primary	Reference			/	
Secondary	1.19 (1.02–1.40)	0.032		/	
Tubal factor existed					
Yes	1.57 (1.24–2.58)	0.017		1.32 (0.87–1.93)	0.081
No	Reference			Reference	
Peak Estrogen level (pg/ml)				/	
< 2500	Reference			/	
[2500–5000)	1.04 (0.86–1.25)	0.686		/	
[5000–7500)	1.38 (1.08–1.75)	0.009		/	
≥ 7500	1.75 (1.27–2.39)	0.001		/	
Endometrial thickness (mm)					
Thin (< 7)	3.04 (1.88–4.93)	< 0.001		5.10 (2.33–10.17)	0.000
Medium (8–13)	1.50 (1.24–1.81)	< 0.001		2.14 (1.09–4.18)	0.027
Thick (≥ 14)	Reference			Reference	
Type of embryos					
Cleavage	1.62 (1.09–2.42)	0.018		1.63 (1.13–2.33)	0.008
Blastocyst	Reference			Reference	
No. of embryos transferred					
1	Reference			Reference	
2	1.22 (0.58–2.57)	0.602		1.24 (0.83–2.96)	0.291
3	1.31 (0.67–2.55)	0.432		1.47 (0.96–3.41)	0.072

*Note* BMI, body mass index; OR, odds ratio; CI, confidence interval

In fresh cycles, adjusted factors were BMI, infertility type, tubal factor, peak estrogen level, endometrial thickness, type of embryos, and number of embryos transferred. In frozen thawed cycles, adjusted factors were BMI, tubal factor, endometrial thickness, type of embryos, and number of embryos transferred

## Discussion

In this retrospective study with a large sample size, no significant association was found between female BMI and the risk of EP in fresh ET cycles after controlled ovarian hyperstimulation (COH) after adjusting for potential confounding factors. In addition, the EP rates among three BMI groups were also comparable in FET cycles.

To date, there has been numerous data identifying risk factors for EP during ART treatment [4, 7, 19]. Whether a correlation exists between female BMI and the risk of EP has been previously discussed. Several Chinese studies of natural conceptions and artificial insemination cycles with donor sperm observed higher EP rates in obese women than those with underweight and normal weight [20, 21], suggesting potential obesity-related tubal dysfunction. In the study including 136,605 clinical pregnancies about the effect of ovarian hyperstimulation protocols on the EP in the United States, Londra et al. also observed that female obesity was associated with an increased risk of EP (BMI ≥ 30 kg/m^2^ vs. < 30 kg/m^2^: adjusted OR 1.33; 95% CI, 1.19–1.47; *P* < 0.001) in fresh autologous ET cycles [8]However, limited by the relatively low incidence of EP and the small proportion of underweight women in the infertile population, the effect of low BMI per se on EP in women was ignored. To our knowledge, Cai et al. firstly proposed a clear association between low BMI and EP during IVF treatment [15]. In the cohort of 16,378 pregnancies derived from fresh and frozen-thawed ET cycles including 2155 underweight women, low BMI (< 18.5 kg/m^2^) was associated with the increased odds of EP (2.92% vs. 2.02%, adjusted OR 1.61; 95% CI, 1.19–2.16; *P* = 0.002, compared with the normal BMI group) but not high BMI (> 24.9 kg/m^2^). They conjectured that underweight women might have a nutrition-related unfavorable uterine environment for embryos, which possibly involved in the underlying mechanism of the higher EP risk. However, Bellver J made a dissent that a suboptimal endometrial milieu would be more likely to hamper embryo implantation or ongoing pregnancy in uterus, resulting in lower implantation rates or higher miscarriage rates rather than implanting in fallopian tube, a tissue non-physiologically suitable for conception [11]. And in women undergoing either oocyte donation or autologous euploid embryo transfer, no significant differences in pregnancy outcomes have been previously reported between underweight and normal weight groups [22–25], which did not support the premise that being underweight might have an adverse effect on the uterine environment. In our study, neither an independent effect of female BMI on EP in fresh cycles after adjusting for potential confounders nor a significant difference in EP rates among the three BMI groups in FET cycles was demonstrated, which differs from the findings of Cai et al. This difference may be attributed to discordant BMI classification criteria and variations in the proportion of the subjects among three groups: 6.2% (1703/27,600) of underweight women, 61.0% (16,840/27,600) of women with normal weight, and 32.8% (9057/27,600) women with overweight/obesity were included in the present study according to the standards of WGOC and ILSI, whereas the proportions were 12.1% (1324/10,930), 82.8% (9047/10,930) and 5.1% (559/10,930) respectively in the study by Cai et al. based on the WHO criteria.

In this study, the EP rates in fresh ET and FET cycles were 2.43% and 2.82% respectively. Our data also identified some risk factors for EP related to IVF treatment, such as tubal factor, elevated peak estrogen levels after COH, endometrial thickness, and stage of embryo for transfer in fresh ET cycles, which concurred with those previously reported [7, 10, 19]. For fresh ET cycles, the results of multivariate regression analysis suggested that the uneven distribution of these factors among the three groups potentially contributed to the higher EP rates in underweight women rather than the independent effect of low female BMI.

Supraphysiologic hormonal milieu after COH in fresh ET cycles is known to increase the risk of EP during IVF treatment, and high estradiol levels may interfere the regulation of tubal physiologic process, which possibly plays a role in the pathophysiological mechanism of tubal EP development after embryos transfer [26]. Wang et al. reported that in fresh ET cycles, high estradiol levels [estradiol on hCG day > 4085 pg/mL] were associated with the increased EP risk (3.4% vs. 2.0%, adjusted OR, 1.99; 95% CI, 1.19–3.35; *P* = 0.009) in women without polycystic ovary syndrome [27]. In our study, lean women accompanied with higher estradiol levels on average yielded higher EP rates in fresh ET cycles, and high peak estradiol levels were related to an increased risk of EP after adjusting potential confounding factors, which was consistent with our previous findings in both tubal infertile women and non-tubal infertile women undergoing fresh ET cycles [7]. For FET cycles without exogenetic high-dose gonadotropin supplementation, hormone levels could be considered close to natural condition, and EP rates were similar among three BMI groups. Taken together, our findings supported the conclusions of Wang et al., and we noticed that lean women appeared to have a sensitivity to ovarian stimulation and develop higher estradiol levels after COH [28]. Thus, when exploring the independent impact of female BMI on EP risk, attention should be paid to eliminating the interference of estradiol levels.

Our findings also confirmed that thin endometrium thickness (EMT) contributed to an increased risk of EP in both fresh cycles and FET cycles, which is in line with multiple existing reports [1, 5]. One potential explanation for the relationship between thin endometrium and higher EP rates is the difference in oxygen tension between the thin endometrium and the fallopian tube. In the thin endometrium with a thin or absent functional layer, the implanting embryos would be closer to the spiral arteries in the basal endometrium layer where the embryos are exposed to higher oxygen concentrations, which may be detrimental to their growth. By contrast, the oxygen tension is relatively lower in the fallopian tube [29]. Consistent with many past studies, this study also presented similar higher EP rates for cleavage-stage embryos compared to blastocyst transfer [7, 9, 30]. Several speculations have been proposed: transferring blastocysts into the uterine cavity seems closer to the physiological state than that of cleavage-stage embryos, requiring a shorter interval for further development before implantation; the size of a blastocyst is larger than that of a cleavage-stage embryo, all of which may reduce the chance of embryos migrating to the fallopian tube [9, 31].

The strength of the current study is the large sample size with the adequate number of underweight women in a single center. Given that the characteristics and distribution of BMI vary by race and region, BMI categories were based on the recommendations on cut-off points of BMI in Chinese adults by WGOC. Therefore, the results were more suitable to provide counseling and guidance for Chinese women undergoing IVF treatment. And the independent effect of female BMI on EP development was discussed in fresh ET and FET cycles respectively. It must be acknowledged that this study has some following limitations. First, as a retrospective study, some potential confounders, such as the volume of transfer fluid, transfer depth and smoking habits, were not available in our database. Second, when investigating the association between development stages of embryos transferred and the occurrence of EP, our study mainly focused on the comparison between cleavage-stage embryos and blastocysts without differentiating the specific stage of a certain blastocyst. In addition, this study spans a long duration over ten years when IVF technology has progressed rapidly, and thus there might be potential biases related to the evolution of IVF behind the results. More well-designed prospective studies with large cohort are needed to evaluate the effect of female underweight or excess weight on EP development after IVF treatment, and possible underlying mechanisms.

## Conclusion

This study suggested that female BMI might be not associated with the risk of ectopic pregnancy in either fresh ET or FET cycles. It may be too early to say with certainty that female underweight, or overweight/obesity is to blame for the higher risk of EP after IVF treatment.

## Data Availability

The datasets used and/or analyzed during the current study are available from the corresponding author on reasonable request.

## Abbreviations

BMI: Body mass index
EP: Ectopic pregnancy
ET: Embryo transfer
ART: Assisted reproduction techniques
IVF: In vitro fertilization
ICSI: Intracytoplasmic sperm injection
GnRH: Gonadotropin-releasing hormone
OHSS: Ovarian hyperstimulation syndrome
FET: Frozen-thawed embryo transfer
COH: Controlled ovarian hyperstimulation
hCG: Human chorionic gonadotropin

## References

[1] Rombauts L, McMaster R, Motteram C, Fernando S (2015). Risk of ectopic pregnancy is linked to endometrial thickness in a retrospective cohort study of 8120 assisted reproduction technology cycles. Hum Reprod (Oxford England).

[2] Acharya KS, Acharya CR, Provost MP, Yeh JS, Steward RG, Eaton JL, Muasher SJ (2015). Ectopic pregnancy rate increases with the number of retrieved oocytes in autologous in vitro fertilization with non-tubal infertility but not donor/recipient cycles: an analysis of 109,140 clinical pregnancies from the society for assisted Reproductive Technology registry. Fertil Steril.

[3] Liu X, Qu P, Bai H, Shi W, Shi J (2019). Endometrial thickness as a predictor of ectopic pregnancy in 1125 in vitro fertilization-embryo transfer cycles: a matched case-control study. Arch Gynecol Obstet.

[4] Anzhel S, Mäkinen S, Tinkanen H, Mikkilä T, Haltia A, Perheentupa A, Tomás C, Martikainen H, Tiitinen A, Tapanainen JS, Veleva Z (2022). Top-quality embryo transfer is associated with lower odds of ectopic pregnancy. Acta Obstet Gynecol Scand.

[5] Liu H, Zhang J, Wang B, Kuang Y (2020). Effect of endometrial thickness on ectopic pregnancy in frozen embryo transfer cycles: an analysis including 17,244 pregnancy cycles. Fertil Steril.

[6] Fang C, Huang R, Wei L-N, Jia L. Frozen-thawed day 5 blastocyst transfer is associated with a lower risk of ectopic pregnancy than day 3 transfer and fresh transfer. Fertil Steril 2015, 103. 10.1016/j.fertnstert.2014.11.023 25542820

[7] Bu Z, Xiong Y, Wang K, Sun Y (2016). Risk factors for ectopic pregnancy in assisted reproductive technology: a 6-year, single-center study. Fertil Steril.

[8] Londra L, Moreau C, Strobino D, Bhasin A, Zhao Y (2016). Is the type of gonadotropin-releasing hormone suppression protocol for ovarian hyperstimulation associated with ectopic pregnancy in fresh autologous cycles for in vitro fertilization?. Fertil Steril.

[9] Li Z, Sullivan EA, Chapman M, Farquhar C, Wang YA (2015). Risk of ectopic pregnancy lowest with transfer of single frozen blastocyst. Hum Reprod (Oxford England).

[10] Perkins KM, Boulet SL, Kissin DM, Jamieson DJ (2015). Risk of ectopic pregnancy associated with assisted reproductive technology in the United States, 2001–2011. Obstet Gynecol.

[11] Bellver J (2022). BMI and miscarriage after IVF. Curr Opin Obst Gynecol.

[12] Sermondade N, Huberlant S, Bourhis-Lefebvre V, Arbo E, Gallot V, Colombani M, Fréour T (2019). Female obesity is negatively associated with live birth rate following IVF: a systematic review and meta-analysis. Hum Reprod Update.

[13] Aune D, Saugstad OD, Henriksen T, Tonstad S (2014). Maternal body mass index and the risk of fetal death, stillbirth, and infant death: a systematic review and meta-analysis. JAMA.

[14] Bu Z, Zhang J, Hu L, Sun Y (2020). Preterm Birth in assisted Reproductive Technology: an analysis of more than 20,000 Singleton Newborns. Front Endocrinol.

[15] Cai J, Liu L, Jiang X, Li P, Sha A, Ren J (2021). Low body mass index is associated with ectopic pregnancy following assisted reproductive techniques: a retrospective study. BJOG: An International Journal of Obstetrics and Gynaecology.

[16] Zhou B-F (2002). Predictive values of body mass index and waist circumference for risk factors of certain related diseases in chinese adults–study on optimal cut-off points of body mass index and waist circumference in chinese adults. Biomed Environ Sci: BES.

[17] Wang D, Chu T, Yu T, Zhai J (2022). Is early-follicular long-acting GnRH agonist protocol an alternative for patients with polycystic ovary syndrome undergoing in vitro fertilization?. Reproductive Biology and Endocrinology: RB&E.

[18] Jin Z, Shi H, Bu Z, Guo Y, Su Y, Song H, Huo M, Yang E, Li J, Zhang Y (2021). Live birth rates after natural cycle versus hormone replacement therapy for single euploid blastocyst transfers: a retrospective cohort study. Reprod Biomed Online.

[19] Jwa SC, Seto S, Takamura M, Kuwahara A, Kajihara T, Ishihara O (2020). Ovarian stimulation increases the risk of ectopic pregnancy for fresh embryo transfers: an analysis of 68,851 clinical pregnancies from the japanese assisted Reproductive Technology registry. Fertil Steril.

[20] Pan Y, Zhang S, Wang Q, Shen H, Zhang Y, Li Y, Yan D, Sun L (2016). Investigating the association between prepregnancy body mass index and adverse pregnancy outcomes: a large cohort study of 536†098 chinese pregnant women in rural China. BMJ Open.

[21] Na L, Chen Y, Zhai H, Liao A, Huang D (2018). Effects of maternal body mass index on pregnancy outcome after 8570 artificial insemination cycles with donor’s sperm. Gynecol Endocrinology: Official J Int Soc Gynecol Endocrinol.

[22] Bellver J, Pellicer A, García-Velasco JA, Ballesteros A, Remohí J, Meseguer M (2013). Obesity reduces uterine receptivity: clinical experience from 9,587 first cycles of ovum donation with normal weight donors. Fertil Steril.

[23] Provost MP, Acharya KS, Acharya CR, Yeh JS, Steward RG, Eaton JL, Goldfarb JM, Muasher SJ (2016). Pregnancy outcomes decline with increasing body mass index: analysis of 239,127 fresh autologous in vitro fertilization cycles from the 2008–2010 society for assisted Reproductive Technology registry. Fertil Steril.

[24] Cozzolino M, García-Velasco JA, Meseguer M, Pellicer A, Bellver J (2021). Female obesity increases the risk of miscarriage of euploid embryos. Fertil Steril.

[25] Romanski PA, Bortoletto P, Chung A, Magaoay B, Rosenwaks Z, Spandorfer SD (2021). Reproductive and obstetric outcomes in mildly and significantly underweight women undergoing IVF. Reprod Biomed Online.

[26] Shao R, Feng Y, Zou S, Weijdegård B, Wu G, Brännström M, Billig H (2012). The role of estrogen in the pathophysiology of tubal ectopic pregnancy. Am J Translational Res.

[27] Wang J, Wei Y, Diao F, Cui Y, Mao Y, Wang W, Liu J (2013). The association between polycystic ovary syndrome and ectopic pregnancy after in vitro fertilization and embryo transfer. Am J Obstet Gynecol.

[28] Navot D, Relou A, Birkenfeld A, Rabinowitz R, Brzezinski A, Margalioth EJ (1988). Risk factors and prognostic variables in the ovarian hyperstimulation syndrome. Am J Obstet Gynecol.

[29] Casper RF (2011). It’s time to pay attention to the endometrium. Fertil Steril.

[30] Du T, Chen H, Fu R, Chen Q, Wang Y, Mol BW, Kuang Y, Lyu Q. Comparison of ectopic pregnancy risk among transfers of embryos vitrified on day 3, day 5, and day 6. Fertil Steril 2017, 108. 10.1016/j.fertnstert.2017.05.027 28602476

[31] Mangalraj AM, Muthukumar K, Aleyamma T, Kamath MS, George K (2009). Blastocyst stage transfer vs cleavage stage embryo transfer. J Hum Reproductive Sci.

